# Case Report: severe *Mycoplasma pneumoniae*-associated acute disseminated encephalomyelitis in an adult: challenges in diagnosis and management

**DOI:** 10.3389/fmed.2025.1627241

**Published:** 2025-08-29

**Authors:** Katharina Feil, Benjamin Ruf, Felix Behling, Gabor Kozak, Georg Gohla, Reimer Riessen, Michael Haap, Sven Poli, Florian Hennersdorf, Ulf Ziemann, Annerose Mengel

**Affiliations:** ^1^Department of Neurology and Stroke, University of Tübingen, Tübingen, Germany; ^2^Department of Internal Medicine I, University Hospital Tübingen, University of Tübingen, Tübingen, Germany; ^3^M3 Research Center for Microbiome, Metabolome and Malignome, Faculty of Medicine, University of Tübingen, Tübingen, Germany; ^4^Cluster of Excellence iFIT (EXC 2180) “Image Guided and Functionally Instructed Tumor Therapies,” University of Tübingen, Tübingen, Germany; ^5^Department of Medicine, Medical Intensive Care Unit, University Hospital Tübingen, Tübingen, Germany; ^6^Department of Neurosurgery, University of Tübingen, Tübingen, Germany; ^7^Hertie Institute for Clinical Brain Research, University of Tübingen, Tübingen, Germany; ^8^Department of Diagnostic and Interventional Neuroradiology, University of Tübingen, Tübingen, Germany

**Keywords:** acute disseminated encephalomyelitis (ADEM), *Mycoplasma pneumoniae*, neurointensive care, decompressive hemicraniectomy, intracranial hypertension

## Abstract

We report a rare case of severe *Mycoplasma (M.) pneumoniae*-associated acute disseminated encephalomyelitis (ADEM) in a previously healthy 26-year-old woman. The patient presented with delayed-onset encephalopathy following a mild respiratory infection. Rapid neurological deterioration, signs of transtentorial herniation, and refractory intracranial hypertension necessitated emergency decompressive hemicraniectomy. Diagnostic workup revealed elevated *M. pneumoniae* IgM and borderline PCR positivity. The patient was successfully treated with corticosteroids, intravenous immunoglobulin, and targeted azithromycin. Complete clinical and radiological recovery was achieved. This case emphasizes the need for awareness of severe *M. pneumoniae*-associated CNS manifestations and highlights the challenges in diagnosis and management.

## Introduction

Acute disseminated encephalomyelitis (ADEM) is a monophasic, immune-mediated demyelinating disease of the central nervous system (CNS), primarily affecting children (1). Adult cases are rare and often more severe. *Mycoplasma (M.) pneumoniae*, a common respiratory pathogen, is a recognized but infrequent trigger of ADEM, with CNS involvement in <0.1% of cases (2). *M. pneumoniae* shows seasonal circulation with winter peaks but can occur year-round (3). We describe a rare and severe adult case requiring neurointensive care and neurossurgical intervention, emphasizing diagnostic and therapeutic challenges, particularly in the context of the 2024 *M. pneumoniae* outbreak.

## Case description

**FIGURE 1:**
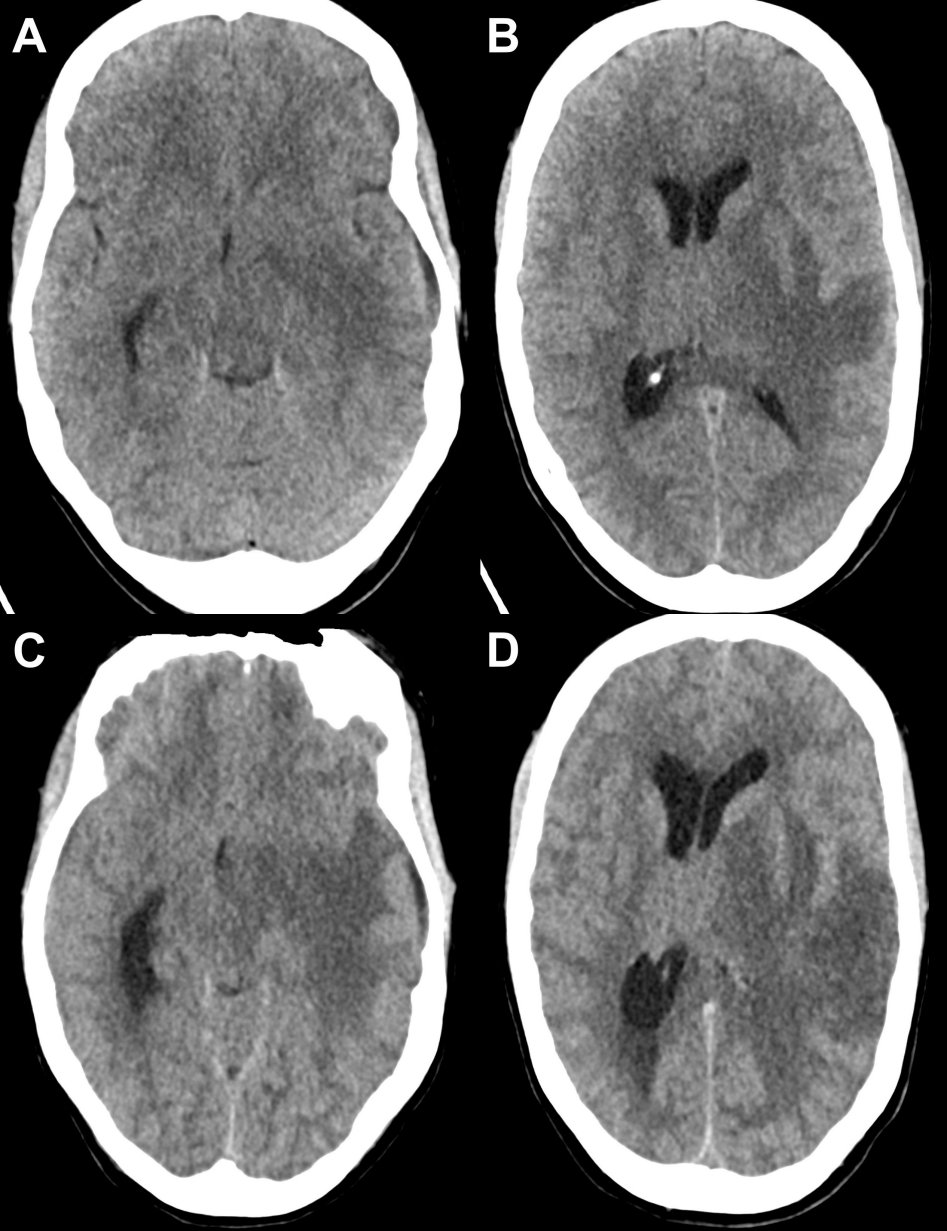
The first row displays two axial slices from the initial CT scan obtained in the emergency setting at the level of the brainstem **(A)** and the thalamus **(B).** Imaging revealed an ill-defined hypodensity in the left temporal region involving both the white matter and the cortical/juxtacortical areas, accompanied by extensive edema, resulting in a rightward midline shift and compression of the left lateral ventricle. No hemorrhage was detected. The second row (**C,D**) shows the follow-up CT scan obtained 2 days later, demonstrating the progression of the lesion, worsening edema, and an increasing mass effect.

In late August 2024, the 26-year-old patient, who was otherwise healthy and on no regular medication, presented with subjective right leg heaviness following a recent upper respiratory tract infection marked by subfebrile fever, cough, and malaise. Neurological examination and duplex sonography to rule out deep vein thrombosis were unremarkable. Her symptoms were initially managed symptomatically with ibuprofen and were clinically attributed to post-viral myalgia. Approximately 3 weeks into the illness, she received a first course of antibiotic therapy with amoxicillin (500 mg three times daily), which was switched to oral azithromycin (500 mg once daily) 2 days prior to hospital admission. Four weeks after symptom onset, her condition acutely deteriorated and she experienced acute consciousness deterioration with meningism and a Glasgow Coma Scale (GCS) of 10. On admission to our neurological intensive care unit (NICU), she displayed global aphasia, severe right-sided hemiparesis, a positive Babinski sign on the right, multimodal neglect, and upbeat nystagmus as well as a spontaneous nystagmus to the left (National Institutes of Health Stroke Scale (NIHSS) 22). Initial non-contrast cranial computed tomography (CT) revealed extensive cortical and subcortical hypodensities and swelling of the left temporal lobe and the left internal as well external capsule and consecutive mass effect and midline shift of 6 mm, but no hemorrhage, ischemia, or vascular abnormalities. Findings were initially interpreted as consistent with herpes encephalitis (Figures 1A, B). Cerebrospinal fluid analysis (CSF) showed pleocytosis (522 cells/μl, 86% mononuclear cells, 14% polymorphonuclear cells), elevated lactate (3.7 mmol/L), and normal glucose (45 mg/dl). Initial treatment included empiric aciclovir, ceftriaxone, and ampicillin. Ampicillin was discontinued on day 3 following negative results for *Listeria*. Polymerase chain reaction (PCR) testing for herpes simplex virus (HSV), varicella-zoster virus (VZV), Epstein-Barr virus (EBV), and *cytomegalovirus* (*CMV*), Human herpesvirus 6 (HHV-6) was negative. Tick-borne encephalitis serum IgG was positive consistent with complete prior vaccination, the last one three years earlier. Comprehensive microbiological testing ruled out bacterial, fungal, and mycobacterial infections. Multiple bacterial cultures from CSF and blood showed no growth after 48 h and remained negative throughout the 5-day incubation period. PCR testing for *Neisseria meningitidis, Streptococcus pneumoniae, Streptococcus agalactiae, Haemophilus influenzae, Escherichia coli K1*, *Listeria monocytogenes, and Mycobacterium tuberculosis* PCR (TB-PCR) was negative. Mycobacterial cultures, incubated for 7 weeks, showed no growth. Further testing for *M. pneumoniae* and *Chlamydia pneumoniae* in CSF was negative, though the *Chlamydia pneumoniae* test was performed outside of accredited diagnostic standards, and sensitivity/specificity data were unavailable. Fungal screening, including *Cryptococcus neoformans*, was negative. Serological testing ruled out syphilis (*Treponema pallidum). I*n addition, testing for Aquaporin-4 (AQP4) and Myelin Oligodendrocyte Glycoprotein (MOG) antibodies was negative. Additional CSF analysis showed a slightly elevated liquor/serum index, consistent with non-specific intrathecal B-cell activation. There was no evidence for neuroborreliosis. In addition, a comprehensive autoimmune antibody panel was performed in serum and CSF, including antibodies for autoimmune and limbic encephalitis (NMDA-R, AMPA-R, GABA-B-R, LGI1, CASPR2, DPPX), and paraneoplastic antibodies (Hu, Yo, Ri, CV2/CRMP5, Ma2/Ta, amphiphysin), all of which were negative. Tracheal secretion PCR showed borderline-positive *M. pneumoniae* (Cq 41.6), corroborated by elevated IgM and IgG titers.

**FIGURE 2:**
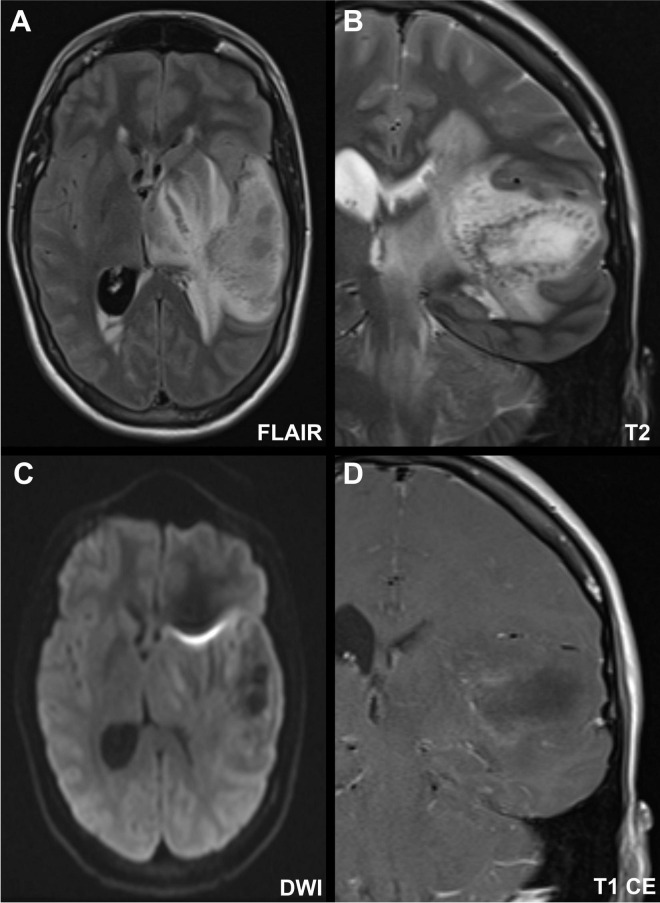
Illustrated are fluid attenuated inversion recovery (FLAIR, A), T2-weighted (B), diffusion weighted imaging (DWI, C), and contrast-enhanced (CE) T1-weighted images from an MRI scan obtained immediately after the progression detected on the CT scan. The images showed a left temporal lesion **(A)** extending **(B)** to the left thalamus, the left cerebral peduncle to the midbrain and the left cerebellar hemisphere. The temporal area is characterized by circularly arranged T2-hypointense, linear-to-punctate, and partially bead-like structures with centrally predominant hyperintense areas. Due to the mass effect, the images demonstrated signs of increased intracranial pressure with hydrocephalus, midline shift, narrowed perimesencephalic cisterns and slight low-lying cerebellar tonsils. There was no evidence of true diffusion restriction, although some regions within the lesion exhibited features suggestive of hypercellularity **(C)**. Following contrast administration, the lesion displayed faint, irregular marginal enhancement resembling an open-ring pattern **(D)**.

The patient’s condition deteriorated over the next 24 h, initially to GCS 6-7, and subsequently to GCS 3. Neurological examination revealed central oculomotor disturbances with skew deviation, characterized by left hypertropia greater than right, incomplete right oculomotor nerve palsy, anisocoria (left > right). Cushing’s triad (bradycardia, hypertension, and deep respiration), suggesting impending transtentorial herniation. Skew deviation was assumed to be of central origin, although secondary ocular misalignment due to the right oculomotor nerve palsy could not be definitively excluded. The clinical signs were consistent with transtentorial herniation, although direct brainstem involvement due to extensive edema could not be entirely ruled out. Repeat CT imaging revealed progressive cerebral edema, brainstem compression, increased midline shift, and signs of herniation (Figures 1C, D). The patient was intubated, and an intracranial pressure (ICP) probe was placed, revealing elevated pressures at around 30 mmHg. Conservative ICP management was initiated including deep sedation, osmotherapy, and head elevation under close monitoring of ICP and Neurological Pupillary Index (NPI). Subsequent contrast-enhanced MRI demonstrated a lesion in the left temporal hemisphere involving the cortical ribbon, characterized by circularly arranged T2-hypointense, linear-to-punctate, and partially bead-like organized structures with centrally predominant hyperintense areas. There was no evidence of hemosiderin deposition or definitive diffusion restriction. The edema extended to the basal ganglia, thalamus, and midbrain, maintaining continuity with a lesion in the left cerebellum. The cerebellar lesion exhibited mixed signal behavior on T2-weighted sequences and appeared hypercellular with corresponding signal reduction on the ADC map. Post-contrast imaging showed no enhancement of the infratentorial lesion, whereas the supratentorial temporal lesion demonstrated faint, irregular, marginal contrast enhancement resembling an open-ring pattern, with the opening directed laterally. The intracranial CSF spaces remained collapsed, with persistent midline shift to the right and signs of CSF obstruction. Infratentorial findings included effacement of the perimesencephalic cisterns and herniation of the cerebellar tonsils through the foramen magnum, consistent with secondary cerebellar tonsillar descent due to elevated intracranial pressure. The imaging findings continued to demonstrate evidence of supra- and infratentorial elevated ICP and CSF obstruction. The rapidly progressive cerebral pathology displayed features that were not characteristic of a single defined entity. Differential diagnoses included encephalitic, inflammatory, or infectious processes, as well as a possible demyelinating condition (Figures 2A–D and Table 1).

**TABLE 1:** Timeline of clinical course, diagnostics, and treatment.

Time (days from onset)	Clinical event	Diagnostics	Treatment
Day 0	Upper respiratory tract infection	—	Symptomatic
Day 21	Amoxicillin begun	—	—
Day 26–27	Azithromycin added	—	—
Day 28 (Thursday)	NICU admission (NIHSS 22, GCS 10)	CT, CSF	Acyclovir, ceftriaxone
Day 29–31	Neurological deterioration (GCS 3, herniation signs)	MRI, ICP monitoring	Conservative ICP management
Day 32 (Monday)	Refractory ICP → Hemicraniectomy	—	Surgery, biopsies
Day 33	Diagnosis: *M. pneumoniae*-associated ADEM	IgM↑, PCR borderline, CSF−	Methylprednisolone, IVIG
Day 34–38	Clinical stabilization	—	Azithromycin (10 days)
Month 2	Clinical recovery, weaning, mobilization	MRI: improving lesions	—
December 2024	Cranioplasty performed	—	—
May 2025 (month 8)	Full neurological recovery (NIHSS 0)	MRI: near-complete resolution	

With the failure of maximal conservative measures to control the elevated ICP exceeding 20 mmHg and the need for further diagnostic clarification via brain biopsy, an emergency decompressive hemicraniectomy was performed. Intraoperatively, multiple biopsies were obtained from the left temporal lobe. Histopathology revealed reactive astrocytosis in gray matter without evidence of neoplasia or infection. The absence of white matter in the probe limited confirmation of demyelination.

Taking all together, the clinical findings and clinical course, neuroimaging and other diagnostic studies, were strongly suggestive of *M. pneumoniae*-associated ADEM. The patient was then further treated with high-dose methylprednisolone (1 g/day for 5 days) and intravenous immunoglobulin (2 g/kg over 4 days) to address the presumed autoimmune component of ADEM. Given the serological confirmation of *M. pneumoniae*, azithromycin was introduced on day 5 of admission at a dose of 500 mg intravenously once daily and continued for 10 days as targeted antimicrobial therapy.

Serial MRI evaluations demonstrated progressive improvement in the extensive T2 hyperintense FLAIR abnormalities in the left hemisphere, initially involving the basal ganglia, mesencephalon, pons, and left cerebellum indicating a favorable radiological response to therapy. By mid-October, she was extubated, in the further course alert, and orientated, though with persistent right-sided motor deficits. The patient had no observed epileptic seizures. During the episode of decreased consciousness, antiseizure treatment was initiated under the differential diagnosis of focal status epilepticus but was discontinued within a few days. After sedation weaning, continuous EEG monitoring showed no epileptiform activity. Cranioplasty was successfully performed in December 2024 with an uneventful postoperative course. No formal neuropsychological assessment was performed during the acute hospital stay. During subsequent inpatient neurorehabilitation, neuropsychological testing revealed transient short-term memory deficits according to the patient’s own account. At the latest follow-up approximately 8 months after the acute event, MRI showed complete resolution of the previous signs of herniation. Clinically, the patient demonstrated full neurological recovery (NIHSS 0, mRS 0). No further immunomodulatory treatment was required. The patient has since returned to her previous employment after a gradual reintegration period.

## Discussion

This case highlights rare but severe neurological complications of *M. pneumoniae* infection in adults. While *M. pneumoniae* is a common cause of atypical pneumonia, central nervous system (CNS) manifestations such as ADEM are exceedingly rare, occurring in less than 0.1% of cases (1). Surveillance data indicate that *M. pneumoniae* activity has resumed typical seasonal circulation following the COVID-19 pandemic. Population-level monitoring from British Columbia, Canada, showed that detection rates increased after implementation of a syndromic nucleic acid amplification test panel - a multiplex PCR assay detecting multiple respiratory pathogens in a single run - while overall incidence remained within expected seasonal levels (3). *M. pneumoniae* is a recognized trigger for autoimmune diseases via molecular mimicry, immune modulation, and inflammatory responses (2). ADEM has an incidence of approximately 8 per 1.000.000 people per year. Most cases occur in children, with an average age of 5–8 years (4–6). As ADEM is an uncommon illness in adults, the precise incidence in adults is unknown (7). Seasonal variation has been observed, with higher incidence during winter and spring, coinciding with peaks in infections (4). Although the precise pathogenesis is not completely understood, ADEM is an autoimmune demyelinating disease of the CNS caused by an inflammatory reaction in the brain and spinal cord. The onset is typically acute and often rapidly progressive, with multifocal neurological deficits (8). ADEM is usually triggered by environmental stimuli in genetically susceptible individuals. One proposed mechanism involves molecular mimicry, where myelin autoantigens such as myelin basic protein, proteolipid protein, and myelin oligodendrocyte protein share antigenic determinants with an infecting pathogen. Antiviral antibodies or cell-mediated responses cross-react with these antigens, resulting in widespread CNS demyelination (9). Its expanding clinical phenotype increasingly overlaps with MOG antibody-associated disorders. A population-based study from Minnesota reported the prevalence of ADEM without MOG antibodies at 3.3 per 100.000 and MOG-associated demyelination prevalence at 1.9 per 100.000 (10).

Clinical, radiographic, and pathological features of ADEM can mimic multiple sclerosis (MS) and experimental autoimmune encephalomyelitis in animal models (1, 10). Although ADEM is typically monophasic, recurrent demyelinating episodes in children or adults may lead to a diagnosis of MS (1, 11). ADEM is part of a continuum of CNS demyelinating disorders, including MS, acute hemorrhagic leukoencephalitis, transverse myelitis, and optic neuritis. ADEM has been associated with several viral infections - such as measles, mumps, rubella, VZV, *EBV*, CMV, and HSV - as well as bacterial infections, including *M. pneumoniae*.(1) Pathophysiologically, *M. pneumoniae*-associated ADEM likely involves a combination of direct microbial invasion and immune-mediated mechanisms, including molecular mimicry and bystander activation. These processes lead to widespread demyelination, primarily in the CNS white matter (12).

Diagnosing ADEM remains challenging due to the absence of specific biomarkers (6, 8). The diagnosis is supported by the rapid progression of encephalopathy, multifocal neurological deficits, characteristic MRI findings, and the exclusion of alternative diagnoses (6, 8). Brain MRI typically reveals large, poorly demarcated, hyperintense lesions on T2/FLAIR sequences, predominantly affecting white matter, deep gray matter, brainstem, and cerebellum. Black hole lesions on T1-weighted MRI, indicative of chronic inflammation in MS, are not typical in ADEM.(11) In our patient, imaging revealed multifocal supra- and infratentorial lesions of varying sizes, ranging from punctate to large flocculent lesions, with areas of open-ring or absent contrast enhancement. The lesions involved the deep white matter as well as the juxtacortical white matter–gray matter interface. A preceding infection and abnormal CSF findings, such as mild lymphocytic pleocytosis and elevated protein, support the diagnosis but are not required. Diagnostic certainty is often delayed due to the time required for serological and CSF analyses, including tests for MOG and aquaporin-4 antibodies, as well as CSF oligoclonal bands. In our case, the patient exhibited a remarkably elevated CSF cell count, which is atypical for “normal” autoimmune disorders such as ADEM, where pleocytosis is usually mild to moderate. This finding raises important questions regarding the underlying pathophysiology. A high CSF cell count may indicate an additional infectious or inflammatory component, while no specific pathogen was identified in the CSF. Further, the delayed onset of neurological symptoms highlights the complexity of identifying *M. pneumoniae*-associated ADEM. It raises questions about whether *M. pneumoniae* was the primary trigger or a secondary infection following an earlier viral illness. However, the presence of elevated *M. pneumoniae* IgM strongly supports a direct association. Despite these uncertainties, the clinical, radiological, and serological findings collectively supported ADEM as the primary diagnosis.

Immunosuppression is the cornerstone of ADEM management. High-dose corticosteroids, such as methylprednisolone or dexamethasone, remain first-line treatment, followed by a 3–6 week taper of oral prednisolone (11, 13, 14). Evidence suggests that methylprednisolone is superior to dexamethasone in achieving favorable outcomes (13). Rapid tapering (<3 weeks) is associated with higher relapse rates and poorer outcomes, (6) for cases unresponsive to corticosteroids, alternative therapies such as intravenous immunoglobulin (IVIG, 0.4 g/kg intravenously daily for 5 days) and/or plasmapheresis, have shown efficacy. Some evidence suggests that a combination of corticosteroids and IVIG may be beneficial, especially in patients with an initial poor response to corticosteroids (15). In our case, the aggressive disease course necessitated early immunomodulatory therapy with a combination of corticosteroid pulse therapy and IVIGs, and surgical intervention with decompressive craniectomy alleviated life-threatening ICP crises. Antimicrobial treatment with azithromycin targeted the underlying *M. pneumoniae* infection, further contributing to neurological recovery, and achieving an excellent outcome.

Compared to pediatric ADEM, where full recovery occurs in 50%–76% of cases (11), adult cases often follow a more severe clinical course, with complete recovery in only 10%–46% (5, 6, 11). Mortality rates in fulminant ICU-treated patients range from 8%–25% (16). Recovery from ADEM occurs typically within 1–6 months, though residual deficits are more common in adults (11).

## Conclusion

This case highlights the importance of recognizing *M. pneumoniae* as a potential cause of severe neurological manifestations in adult patients. The unusually high prevalence of *M. pneumoniae* infections in 2024 further emphasizes the need for increased clinical vigilance, as outbreaks may lead to a rise in associated complications such as ADEM. Prompt diagnosis, aggressive immunotherapy, and NICU treatment including surgical intervention in cases of refractory ICP elevation are essential for optimizing outcomes. Future research should focus on elucidating the pathophysiology and improving therapeutic strategies for *M. pneumoniae*-associated ADEM.

## Patient perspective

The patient reported being unaware of the potential severity of respiratory infections. After a prolonged ICU and rehabilitation stay, she made a full neurological recovery and has since resumed her normal life and employment. She expressed deep gratitude for the intensive care and neurorehabilitation provided.

## Data Availability

The original contributions presented in this study are included in this article/supplementary material, further inquiries can be directed to the corresponding author.
